# PReS-FINAL-2048: Treatment with methotrexate plus leflunomide for juvenile idiopathic arthritis

**DOI:** 10.1186/1546-0096-11-S2-P61

**Published:** 2013-12-05

**Authors:** E Quesada-Masachs, C Modesto

**Affiliations:** 1 Rheumatology, Hospital Universitario Vall d'Hebron, Barcelona, Spain

## Introduction

Methotrexate (MTX) is the agent of first choice for the treatment of children with Juvenile Idiopathic Arthritis (JIA). Leflunomide (LFN) has demonstrated to be an effective alternative to MTX. There is a lack of evidence regarding the advantages of combined treatment of MTX plus LFN in JIA.

## Objectives

To evaluate the safety and effectiveness of the combined therapy with Methotrexate (MTX) and Leflunomide (LFN) in patients with Juvenile Idiopathic Arthritis (JIA) in clinical practice.

## Methods

We conducted a retrospective descriptive study of patients with JIA visited in a single Unit of Pediatric Rheumatology who had been treated with the combination of MTX plus LFN. All patients were classified according to the International League of Associations for Rheumatology (ILAR) criteria. Included data were: demographics, JIA subtype, reason for starting combined treatment, time on treatment, withdrawals, causes of discontinuation, efficacy and safety (both assessed at baseline and every 6 months during 2 years of treatment). Safety was evaluated analyzing adverse events (AE) based on clinical and physical findings and laboratory values. Ocular effectiveness was assessed grading uveitis according to the Standardization of Uveitis Nomenclature Working Group (SUN) recommendations. Articular effectiveness was assessed by means of: pediatric core set variables with exception of C-HAQ, Protein-C-reactive, JADAS scores 10, 27 and 71, and applying the criteria of minimal disease activity, 30% improvement, inactivity and remission.

## Results

Nineteen patients (16 female, 3 male) were included: 12 oligoarthritis (63%), 4 polyarthritis (21%), 2 psoriatic arthritis (11%) and 1 undifferentiated arthritis (5%). The mean age at diagnosis was 57 months (4.75 years ± 3.65) and at initiation of combined treatment was 112 months (9.37 years ± 4.12).The mean duration of MTX+LFN treatment was 28.6 months ± 31.6 (range 5-144). An improvement was observed in each variable studied. JADAS scores had improved a 60% at last follow up compared to the baseline. The best rates of improvement were observed at 18 months of treatment: 91.7% improved at least 30%, 83.3% met criteria for minimal disease activity, 83.3% for inactivity and 66.7% for clinical remission. Eight children had uveitis and all of them were in clinical remission according to the SUN criteria at last follow up. Overall, 28 AE were reported. There were no serious AE. The most frequently reported AE was upper respiratory tract infection. One of the two DMARD was stopped in 11 patients (58%) due to AE in 4 children, inefficacy or loss of efficacy in 3 and clinical remission in 4. Gastrointestinal intolerance was the more common cause of discontinuation of one of the two DMARD. Among patients who withdrew the combined treatment 4 switched to anti-TNF therapy and 7 continued monotherapy with MTX or LFN. Eight children are still on MTX plus LFN.

## Conclusion

In our patients with JIA the combined therapy of MTX plus LFN was well-tolerated and proved to be effective. Most of the patients experienced a substantial improvement either articular or ocular. AE were generally mild. LFN plus MTX could be a safe and effective alternative for patients with JIA who do not respond to MTX or LFN in monotherapy.

## Disclosure of interest

None declared.

